# Risk Factors and Clinical Outcomes of Deep Surgical Site Infections in Trauma Patients: A National Database Analysis

**DOI:** 10.3390/healthcare13151808

**Published:** 2025-07-25

**Authors:** Musaed Rayzah

**Affiliations:** Department of Surgery, College of Medicine, Majmaah University, P.O. Box 66, Al-Majmaah 11952, Riyadh, Saudi Arabia; m.rayzah@mu.edu.sa

**Keywords:** trauma surgery, surgical site infections, abdominal surgery, risk factors, complications, injury severity score

## Abstract

**Background:** Deep surgical site infections (SSIs) represent a serious complication following abdominal trauma surgery; however, comprehensive risk factor analysis in large trauma populations remains limited. Although surgical site infections are recognized as preventable complications, little is known about the specific risk factors and clinical outcomes associated with deep SSIs in trauma patients at the national level. **Methods:** A retrospective cohort study analyzed data from the National Trauma Data Bank from 2020–2022, including 1,198,262 trauma patients with complete demographic, injury severity, and surgical procedure data. Deep SSI development, length of hospital stay, intensive care unit utilization, duration of mechanical ventilation, discharge disposition, and in-hospital mortality were assessed. Multivariate logistic regression was used to identify independent risk factors and quantify associations between patient characteristics and deep SSI occurrence. **Results:** Deep SSIs occurred in 601 patients (0.05%). Affected patients were younger (median 41 vs. 54 years, *p* < 0.001), predominantly male (73.7% vs. 61.8%, *p* < 0.001), and exhibited higher injury severity scores (median 17.0 vs. 5.0, *p* < 0.001). Major abdominal surgery was the strongest independent predictor (OR 3.08, 95% CI: 2.21–4.23, *p* < 0.001), followed by injury severity score (OR 1.05, 95% CI: 1.04–1.06, *p* < 0.001) and ICU length of stay (OR 1.04 per day, 95% CI: 1.03–1.05, *p* < 0.001). Patients with deep SSIs demonstrated dramatically increased hospital stays (89.5% vs. 4.5% exceeding 21 days, *p* < 0.001), reduced home discharge rates (28.5% vs. 48.9%, *p* < 0.001), and higher mortality (4.2% vs. 1.2%, *p* < 0.001). **Conclusions:** Major abdominal surgery and injury severity are primary risk factors for deep SSIs in trauma patients, with profound impacts on clinical outcomes and healthcare resource utilization. These findings highlight the importance of targeted prevention strategies for high-risk trauma patients undergoing major abdominal procedures and emphasize the significant burden that deep SSIs place on healthcare systems.

## 1. Introduction

Surgical site infections constitute a formidable challenge in surgery, with deep surgical site infections representing the most clinically consequential subset of preventable complications [1,2]. Deep infections penetrate fascial planes and muscle layers, necessitating surgical debridement and prolonged antimicrobial therapy, whereas superficial infections typically respond to conservative management [3]. This distinction becomes particularly salient in trauma populations, where physiological compromise, contaminated wounds, and emergent surgical intervention create an environment conducive to infectious complications [4,5,6,7].

Surgical site infections affect 2–5% of surgical patients, despite advances in perioperative protocols and infection prevention strategies [8]. Trauma patients demonstrate disproportionately elevated infection rates compared to elective surgical cohorts owing to the emergent nature of procedures that preclude preoperative optimization, including nutritional support, infection screening, and hemodynamic stabilization. Additionally, traumatic injuries frequently mandate complex, multisystem surgical interventions with extended operative duration and potential contamination from gastrointestinal or genitourinary sources.

Infectious complications in trauma extend beyond surgical site infections to encompass pneumonia and sepsis, which significantly impacts patient outcomes. Patients with blunt cerebrovascular injuries demonstrate increased odds of developing pneumonia during hospitalization [9], while sepsis presents with significant attributable mortality and excess length of stay [10]. The presence of comorbidities, injury severity, and prolonged hospital stay are critical risk factors for the development of infectious complications [9,11]. Trauma patients with severe injuries or those requiring prolonged mechanical ventilation are particularly susceptible to infections [11]. Multiple studies from Saudi Arabia highlight significant challenges accompanying surgical site infections across different regions and surgical specialties. A multi-regional cross-sectional study revealed that 49.1% of 375 surgical patients across Saudi Arabia’s five regions displayed poor awareness of surgical site infections, with only 32.8% receiving healthcare worker education and 42% learning from external sources, including social media, indicating substantial gaps in patient education [12]. In the Eastern region, a retrospective cohort study reported a 4.7% SSI rate following cesarean sections, identifying diabetes mellitus (OR = 10.76) and surgery duration >1 h (OR = 3.54) as significant independent risk factors [13]. Meanwhile, a retrospective study documented concerning SSI rates of 15.45% and 9.29% following cholecystectomy/appendectomy in the Al-Jouf region during 2021–2022, with MRSA and Klebsiella pneumoniae as predominant pathogens, although implementation of enhanced infection control measures showed promising reductions in infection rates [14].

The timing of infection complications varies by surgical site infection type, with pneumonia and sepsis occurring at different intervals post-operatively [15]. Antibiotic prophylaxis protocols, including appropriate agent selection, dosing, and duration, significantly influence SSI development, with standardized protocols demonstrating substantial infection rate reductions in trauma surgery [16]. Understanding the relationship between patient factors (age, sex), injury characteristics (severity, mechanism), surgical complexity (organ involvement, procedure type), and postoperative variables (ICU stay duration) with SSI incidence remains crucial for developing targeted prevention strategies and improving patient outcomes.

A contemporary understanding of deep surgical site infection risk factors encompasses several key patient-related and procedural variables. Male sex, smoking history, and obesity (BMI > 26.0) were significant demographic risk factors, with open injuries conferring additional susceptibility [17,18]. Surgical variables, particularly operative duration exceeding 60 min and wound classification ≥ 2, demonstrate robust associations with deep surgical site infection development [17,18]. Demographic factors, such as age and sex, require consideration when analyzing trauma outcomes, as older adults demonstrate increased vulnerability to complications due to physiological factors [19]. The microbial landscape is dominated by *Staphylococcus aureus*, including methicillin-resistant strains, with polymicrobial infections presenting particular therapeutic challenges [20,21].

Prevention strategies encompass comprehensive multiphase interventions. Preoperative chlorhexidine-based skin antisepsis demonstrates superior efficacy compared with povidone-iodine solutions, while smoking cessation and weight optimization represent modifiable risk factors [18,22]. Intraoperative strategies emphasize sterile technique adherence, minimized operating room traffic, and surgical duration optimization [23,24]. Postoperative surveillance and empirical antimicrobial therapy tailored to institutional microbial profiles completed the prevention framework [21,25].

Contemporary challenges complicate infection-prevention efforts. Antimicrobial-resistant organisms necessitate the continuous reevaluation of prophylactic strategies [22]. Variability in surgical site infection classification and reporting standards impedes the development of universally applicable prevention algorithms [22,26]. Individualized risk stratification that incorporates patient-specific factors, injury characteristics, and institutional resources remains critical for optimizing prevention strategies [24].

The National Trauma Data Bank serves as a critical resource for examining rare complications in trauma populations, encompassing detailed demographic, injury, procedural, and outcome data from over 900 participating trauma centers nationwide. This vast repository provides a unique opportunity to investigate infectious complications that are crucial for improving trauma-care outcomes. However, specific infectious complications and their risk determinants have not been extensively studied, despite the capacity of the NTDB for comprehensive risk-adjusted analyses [27]. The variability in analytical methodologies across NTDB studies presents challenges for standardized research approaches [27]. Standardizing these analytical techniques could enhance the research quality and provide more reliable insights into infectious complications. This methodological consideration is particularly important when examining rare outcomes that require robust statistical power for meaningful analysis. Identifying and addressing infectious complications with high attributable mortality and excess length of stay represents a crucial opportunity for performance improvement in trauma care [10].

The present investigation leveraged the National Trauma Data Bank to examine deep surgical site infection risk factors and outcomes in the largest trauma population analyzed to date. Our objectives were threefold: first, to identify demographic, injury-related, and treatment-related risk factors through robust multivariate analysis; second, to quantify clinical outcomes and healthcare resource utilization associated with these infections; and third, to provide evidence-based recommendations for infection prevention and risk stratification.

## 2. Methodology

### 2.1. Data Source and Population

This retrospective cohort study analyzed data from the National Trauma Data Bank (NTDB) from 2020–2022, a comprehensive registry maintained by the American College of Surgeons Committee on Trauma in the United States. The study population was restricted to patients with complete demographic data, injury severity scores, and surgical procedure information. Access to NTDB data requires formal application to the American College of Surgeons Committee on Trauma.

### 2.2. Classifications

Deep surgical site infections were defined as infections involving deep soft tissues (fascial and muscle layers) of the incision. Major abdominal surgical procedures were identified using ICD-10-PCS codes through systematic pattern-matching. The procedures included exploratory laparotomy, gastrointestinal resections, hepatobiliary procedures, and splenectomy. A composite major abdominal surgery indicator was constructed using the gastrointestinal, hepatobiliary, and splenic procedure codes. Patient-level data were aggregated using maximum values for binary indicators and temporal sequencing for multiple procedures. Body region-specific datasets were created for six anatomical regions based on AIS coding: head/neck, face, chest, abdomen, extremities, and external regions. Isolated injury was defined as trauma to one region, with all other regions assigned an AIS severity < 3 (none, minor, or moderate injuries only). This threshold excluded cases with serious, severe, critical, or maximum injuries in non-target regions, while permitting minor concurrent injuries. Hospital complications were identified from NTDB event-level data and transformed into patient-level binary indicators. A total of 29 complications were categorized as infectious (n = 10), cardiovascular (n = 4), respiratory (n = 3), neurological (n = 3), renal (n = 1), wound/skin (n = 3), procedural (n = 3), and substance-related (n = 2). Event-level records were converted to patient-level records using maximum values to ensure that the presence of complications was retained when multiple records existed per patient.

### 2.3. Outcomes

The primary outcome was the development of deep surgical site infection during hospitalization, as defined by NTDB coding standards. Secondary outcomes included: (1) hospital length of stay categorized as ≤7 days, 8–14 days, 15–21 days, and >21 days; (2) intensive care unit length of stay; (3) duration of mechanical ventilation; (4) discharge disposition, including home discharge, skilled nursing facility, rehabilitation facility, other acute care hospital, other healthcare facility, left against medical advice, and in-hospital mortality; (5) prolonged mechanical ventilation defined as >48 h; and (6) in-hospital mortality.

### 2.4. Statistical Analysis

Univariate analysis was used to compare patient characteristics using Chi-square tests for categorical variables and Mann–Whitney U tests for continuous variables. Multivariate logistic regression identified independent risk factors for deep surgical site infection, including age, sex, injury severity score, trauma mechanism, major organ injuries, surgical procedures, and ICU length of stay. Variables with *p* < 0.05 in univariate analysis were included in the multivariable model. A complete case analysis was performed, and race/ethnicity were excluded from the multivariable analysis due to missing data. Statistical significance was set at *p* < 0.05. All analyses were conducted using Stata version 17.0 (StataCorp LLC, College Station, TX, USA). This study used de-identified data from the National Trauma Data Bank (NTDB). As this research involved the analysis of existing de-identified datasets, institutional review board approval was not required, in accordance with federal regulations for human subject research.

## 3. Results

### 3.1. Patient Demographics and Injury Characteristics

Analysis of 1,198,262 trauma patients revealed distinct demographic and injury patterns associated with the development of deep surgical site infections (Table 1). Patients developing deep SSIs exhibited a significantly younger age distribution, with a median age of 41 years (IQR: 30–56) compared to 54 years (IQR: 33–71) among those without infection (*p* < 0.001). Male predominance was markedly pronounced in the deep SSI cohort, comprising 73.7% of the cases vs. 61.8% in the uninfected group (*p* < 0.001). Racial composition demonstrated notable disparities, with Black/African American patients disproportionately represented among deep SSI cases (26.8% vs. 17.0%, *p* < 0.001), while White patients comprised a smaller proportion (57.2% vs. 69.2%).

Injury severity metrics revealed substantial differences between the groups. Patients with deep SSIs sustained significantly more severe trauma, as evidenced by a median injury severity score of 17.0 (IQR: 10.0–22.0) compared to 5.0 (IQR: 4.0–10.0) in uninfected patients (*p* < 0.001). The mechanism of injury patterns differed considerably, with motor vehicle crashes predominating among patients with deep SSIs (60.7% vs. 35.2%). Conversely, falls represented a smaller proportion of deep SSIs (9.5% vs. 40.9%). Violence-related mechanisms were strongly associated with the development of deep SSIs, including firearm injuries (14.1% vs. 4.0%, *p* < 0.001) and assault-related trauma (15.1% vs. 11.6%, *p* < 0.001). Penetrating injury mechanisms occurred nearly twice as frequently in patients who developed deep SSIs (17.6% vs. 9.0%, *p* < 0.001).

### 3.2. Clinical Presentation and Surgical Interventions

Patients developing deep SSIs presented with evidence of physiological compromise and required substantially more complex surgical management (Table 2). Hemodynamic instability was evident through a significantly lower median systolic blood pressure at presentation (124 mmHg [IQR: 103–142] vs. 137 mmHg [IQR: 121–155], *p* < 0.001). Neurological impairment was more prevalent, with severe Glasgow Coma Scale scores (3–8) documented in 12.1% of deep SSI patients compared to 3.6% in uninfected patients (*p* < 0.001).

The surgical intervention patterns revealed striking differences in procedural complexity and anatomical involvement. Major abdominal surgery was performed in 22.1% of the patients who developed deep SSIs vs. only 1.8% of those who remained uninfected (*p* < 0.001), representing a 12-fold increase in surgical complexity. Gastrointestinal procedures demonstrated the strongest association with the subsequent development of deep SSIs. Bowel resection procedures were performed in 6.5% of patients with deep SSIs compared with 0.22% of uninfected patients (*p* < 0.001). Similarly, colectomy was required in 4.7% vs. 0.11% (*p* < 0.001), and small bowel surgery was required in 2.2% vs. 0.09% (*p* < 0.001).

Organ-specific injury repairs were substantially more frequently in patients who developed deep SSIs. Colon injury repair was performed in 7.3% of deep SSI cases vs. 0.47% of uninfected cases (*p* < 0.001). Small bowel injury repair occurred in 6.5% vs. 0.48% (*p* < 0.001), and liver injury repair occurred in 6.3% vs. 1.7% (*p* < 0.001). The incidence of deep SSIs remained temporally stable throughout the study period from to 2020–2022 (*p* = 0.3), indicating no significant impact from COVID-19 pandemic-related factors.

Mortality outcomes revealed significant increases in adverse events in patients with deep SSIs. In-hospital mortality was elevated by 3.5-fold (4.2% vs. 1.2%, *p* < 0.001). Additionally, the rates of leaving against medical advice increased substantially (4.2% vs. 1.2%, *p* < 0.001), potentially reflecting patient frustration with prolonged hospitalization or socioeconomic barriers to extended care.

### 3.3. Risk Factor Analysis

Multivariate logistic regression analysis of 123,833 patients identified several independent predictors of the development of deep SSIs (Table 3). Injury severity remained a powerful predictor after adjustment for confounding variables, with each point increasing in injury severity score, conferring a 5% increase in deep SSI risk (OR 1.05, 95% CI: 1.04–1.06, *p* < 0.001) (Table 4).

The mechanism of injury demonstrated a significant independent association with the development of deep SSIs. Compared to motor vehicle crashes as the reference category, falls were associated with a substantially reduced deep SSI risk (OR 0.28, 95% CI: 0.18–0.44, *p* < 0.001). Other injury mechanisms, including motorcycle crashes, firearm injuries, and cut/pierce mechanisms, showed no statistically significant associations in the adjusted analysis.

Major abdominal surgery emerged as the strongest independent predictor of deep SSI development, conferring a three-fold increase in risk (OR 3.08, 95% CI: 2.21–4.23, *p* < 0.001). Prolonged intensive care unit length of stay demonstrated a dose–response relationship, with each additional day associated with a 4% increase in deep SSI risk (OR 1.04, 95% CI: 1.03–1.05, *p* < 0.001). Notably, several variables that demonstrated significant associations in the univariate analysis, including sex, trauma type, and specific organ injuries, lost statistical significance after multivariable adjustment, suggesting confounding by injury severity and surgical complexity.

## 4. Discussion

Deep surgical site infections constitute a serious complication in trauma surgery, penetrating fascial planes and muscle layers and necessitating surgical debridement and prolonged antimicrobial therapy. This study demonstrates three principal findings: deep infections predominantly afflict patients sustaining high-energy trauma requiring complex abdominal interventions; major abdominal surgery constitutes the most potent independent predictor of deep infection development; and these infections precipitate significant healthcare resource consumption, with enduring functional consequences.

The demographic characteristics of patients developing deep site infections fundamentally contradict the established surgical risk stratification frameworks used in elective populations. While conventional surgical cohorts demonstrate increasing infection susceptibility with advancing age due to immunosenescence and comorbidity accumulation [19], trauma patients developing deep infections exhibited a significantly younger median age (41 vs. 54 years). This counterintuitive finding reflects the complex interplay between age, injury mechanism, and surgical complexity inherent in trauma care. Younger patients sustain disproportionately severe trauma through high-energy mechanisms, such as motor vehicle crashes, violence, and falls from height, necessitating extensive surgical reconstruction that increases infection vulnerability, despite physiological resilience.

The predominance of penetrating mechanisms among deep infection cases aligns with established principles regarding contamination potential [17,18], yet quantifies this relationship within contemporary trauma populations. These mechanisms frequently involve gastrointestinal tract violations, introduction of enteric organisms into sterile surgical fields, and creation of polymicrobial infection environments dominated by *Staphylococcus aureus* and Gram-negative enteric bacteria [20,21]. The emergent nature of trauma surgery precludes standard preoperative optimization protocols, such as nutritional support, infection screening, and hemodynamic stabilization, which reduce the infection risk in elective populations. This disparity between trauma and elective surgery may explain why traditional risk factors fail to accurately predict the development of infection in trauma cohorts.

The dose-response relationship between the injury severity score and deep infection risk establishes a framework for evidence-based risk stratification and informed consent discussions. This relationship likely reflects the cumulative impact of physiological derangement, surgical complexity, and prolonged critical care requirements associated with severe trauma. Patients with an ISS > 15 warrant enhanced infection prevention protocols and family counseling regarding complication risks, as supported by the existing literature demonstrating increased infection susceptibility in severely injured patients [9,11]. However, the relatively modest effect size per ISS point suggests that injury severity alone cannot adequately predict infection risk, thereby emphasizing the multifactorial nature of this complication.

The resource consumption patterns associated with deep surgical site infections demonstrate the profound economic burden of this complication, which extends far beyond immediate treatment costs. The finding that 89.5% of infected patients required hospitalization exceeding 21 days compared to only 4.5% of uninfected patients represents a 20-fold increase in prolonged hospitalization, translating to substantial direct and opportunity costs through bed utilization. This pattern assumes particular relevance given ongoing healthcare capacity constraints and the finite nature of trauma center resources. These functional consequences align with established literature demonstrating that infectious complications compromise the long-term quality of life, occupational capacity, and independence in activities of daily living [10]. The societal burden extends beyond healthcare costs to encompass lost productivity, caregiver burden, and reduced quality-adjusted life-years. Understanding these long-term implications is crucial for comprehensive cost-effectiveness analyses of infection-prevention interventions.

Current infection prevention strategies in trauma surgery have evolved significantly; however, their implementation remains inconsistent across trauma centers. The strong association between major abdominal surgery and deep infection development justifies enhanced prevention protocols for patients undergoing gastrointestinal procedures, including the consideration of extended antibiotic prophylaxis duration and intensified postoperative wound surveillance. Current guidelines recommend prophylaxis discontinuation within 24 h, yet trauma patients may benefit from extended coverage, given the contamination risk and physiologic compromise.

The dose–response relationship with injury severity supports risk-stratified prevention approaches, with severely injured patients (ISS > 15) warranting enhanced measures, including chlorhexidine-based skin preparation [18,22], optimized operating room traffic control [23,24], and aggressive nutritional support. However, the implementation of these evidence-based interventions remains variable, similar to the underutilization of epidural analgesia in rib fracture management, despite its proven efficacy. The temporal stability of infection rates throughout 2020–2022 demonstrates trauma system resilience during the COVID-19 pandemic, supporting the effectiveness of established prevention protocols even under extraordinary circumstances.

The limitations of this study are primarily due to the retrospective analysis of a multi-institutional database. The National Trauma Data Bank, a voluntary database contributed to by trauma centers, may not accurately reflect the care provided to all trauma patients in the United States. One consideration is that admission to a Level I trauma center may increase the likelihood of receiving comprehensive infection prevention protocols, possibly leading to an underestimation of infection rates in the broader trauma population. Additionally, the NTDB does not record detailed information regarding specific antibiotic prophylaxis protocols, wound management techniques, or surgeon-specific factors, making it challenging to determine the prevalence of evidence-based prevention strategies. The lack of hospital readmission data in our dataset prevented assessment of this clinically important outcome. The absence of comorbidity data in our analysis represents another significant limitation. The inability to adjust for these important confounding variables may have influenced our risk estimates. Future investigations should prioritize complete demographic data collection to examine potential disparities in infection risk and outcomes, as the social determinants of health significantly influence trauma outcomes [9].

## 5. Conclusions

Deep surgical site infection following trauma represents a preventable complication with catastrophic consequences for patients and the healthcare system. The clinical outcomes associated with deep SSIs are severe, including increases in prolonged hospitalization, ICU length of stay, increased mechanical ventilation requirements, reduced home discharge rates, and elevated mortality. The identification of major abdominal surgery, injury severity, and prolonged critical care as primary risk factors provides actionable targets for prevention efforts. The profound impact on functional outcomes—reducing home discharge rates and increasing prolonged hospitalizations—underscores the critical importance of evidence-based infection prevention protocols in trauma surgery. Although enhanced prevention strategies remain conditionally recommended, the devastating consequences demonstrated in this analysis warrant further implementation. The dose–response relationship between injury severity and infection risk enables risk-stratified prevention approaches for high-risk patients, particularly those undergoing major abdominal procedures or presenting with injury severity scores exceeding 15. These findings support enhanced surveillance and prevention efforts for high-risk trauma patients, while highlighting the urgent need for continued research into modifiable risk factors and optimal prevention strategies to reduce this devastating complication in trauma populations.

## Figures and Tables

**Table 1:** Demographic and injury characteristics by deep surgical site infection status.

Variable	Overall N = 1,198,262	Deep SSI Status		*p*-Value
		No Deep SSI N = 1,197,661	Deep SSI N = 601	
Demographics				
Age (years)	54 (33, 71)	54 (33, 71)	41 (30, 56)	<0.001
Sex				<0.001
Male	740,689 (61.8%)	740,246 (61.8%)	443 (73.7%)	
Race/Ethnicity				<0.001
White	829,280 (69.2%)	828,936 (69.2%)	344 (57.2%)	
Black/African American	203,186 (17.0%)	203,025 (17.0%)	161 (26.8%)	
Asian	26,677 (2.2%)	26,660 (2.2%)	17 (2.8%)	
American Indian/Alaska Native	9546 (0.8%)	9538 (0.8%)	8 (1.3%)	
Hispanic/Latino	106,756 (8.9%)	106,700 (8.9%)	56 (9.3%)	
Injury characteristics				
Injury Severity Score	5.0 (4.0, 10.0)	5.0 (4.0, 10.0)	17.0 (10.0, 22.0)	<0.001
Mechanism of injury				<0.001
Motor vehicle crash	422,261 (35.2%)	421,896 (35.2%)	365 (60.7%)	
Motorcycle crash	60,837 (5.1%)	60,794 (5.1%)	43 (7.2%)	
Fall	489,702 (40.9%)	489,645 (40.9%)	57 (9.5%)	
Struck by/against object	57,073 (4.8%)	57,054 (4.8%)	19 (3.2%)	
Firearm injury	48,500 (4.0%)	48,415 (4.0%)	85 (14.1%)	
Cut/Pierce	1677 (0.1%)	1676 (0.1%)	1 (0.2%)	
Machinery	12,412 (1.0%)	12,408 (1.0%)	4 (0.7%)	
Other transport	73,152 (6.1%)	73,145 (6.1%)	7 (1.2%)	
Natural/Environmental	54 (0.005%)	54 (0.005%)	0 (0.0%)	
Other/Unspecified	14,443 (1.2%)	14,432 (1.2%)	11 (1.8%)	
Trauma type				<0.001
Blunt	1,065,294 (88.9%)	1,064,811 (88.9%)	483 (80.4%)	
Penetrating	108,175 (9.0%)	108,069 (9.0%)	106 (17.6%)	
Burn	1580 (0.1%)	1579 (0.1%)	1 (0.2%)	
Other	4289 (0.4%)	4289 (0.4%)	0 (0.0%)	
Intent of injury				<0.001
Unintentional	1,028,064 (85.8%)	1,027,583 (85.8%)	481 (80.0%)	
Self-Inflicted	15,341 (1.3%)	15,325 (1.3%)	16 (2.7%)	
Assault/Violence	134,188 (11.2%)	134,103 (11.2%)	85 (14.1%)	
Legal intervention	5130 (0.4%)	5124 (0.4%)	6 (1.0%)	
Undetermined	1604 (0.1%)	1600 (0.1%)	4 (0.7%)	

**Table 2:** Clinical parameters and surgical procedures by deep SSI status.

Variable	No Deep SSI (N = 1,197,661)	Deep SSI (N = 601)	*p*-Value
Clinical Parameters			
Systolic BP (mmHg) *	137 (121, 155)	124 (103, 142)	<0.001
GCS score *	15.0 (15.0, 15.0)	15.0 (14.0, 15.0)	<0.001
Severe GCS (3–8)	42,703 (3.6%)	73 (12.1%)	<0.001
Major procedures			
Any major surgery	21,241 (1.8%)	133 (22.1%)	<0.001
High-Risk Procedures			
Colectomy	1328 (0.11%)	28 (4.7%)	<0.001
Bowel resection	2631 (0.22%)	39 (6.5%)	<0.001
Small bowel surgery	1102 (0.09%)	13 (2.2%)	<0.001
Organ Injury Repairs			
Colon injury repair	5587 (0.47%)	44 (7.3%)	<0.001
Small bowel injury repair	5777 (0.48%)	39 (6.5%)	<0.001
Liver injury repair	20,091 (1.7%)	38 (6.3%)	<0.001
Spleen injury repair	16,385 (1.37%)	31 (5.2%)	<0.001

* Median (IQR); all other values = n (%).

**Table 3:** Outcome and procedure variables by deep surgical site infection status.

Variable	Overall N = 1,198,262	Deep SSI Status		*p*-Value
		No Deep SSI N = 1,197,661	Deep SSI N = 601	
Discharge disposition				
Discharge home	585,194 (48.9%)	585,023 (48.9%)	171 (28.5%)	<0.001
Discharge destination				<0.001
Home	585,194 (48.9%)	585,023 (48.9%)	171 (28.5%)	
Skilled nursing facility	291,746 (24.4%)	291,394 (24.3%)	352 (58.6%)	
Rehabilitation facility	8792 (0.7%)	8777 (0.7%)	15 (2.5%)	
Other acute care hospital	9359 (0.8%)	9355 (0.8%)	4 (0.7%)	
Other healthcare facility	22,581 (1.9%)	22,575 (1.9%)	6 (1.0%)	
Left against medical advice	14,797 (1.2%)	14,772 (1.2%)	25 (4.2%)	
Expired	24,462 (2.0%)	24,436 (2.0%)	26 (4.3%)	
Ventilation outcomes				
Prolonged ventilation	29,489 (39.8%)	29,254 (39.7%)	235 (73.2%)	<0.001
Mechanical ventilation days	2.0 (1.0, 4.0)	2.0 (1.0, 4.0)	4.0 (2.0, 11.0)	<0.001
Length of stay				
ICU length of stay (days)	3.0 (2.0, 5.0)	3.0 (2.0, 5.0)	7.0 (4.0, 15.0)	<0.001
Hospital length of stay category				<0.001
≤7 days	666,003 (55.6%)	665,996 (55.6%)	7 (1.2%)	
8–14 days	330,975 (27.6%)	330,953 (27.6%)	22 (3.7%)	
15–21 days	138,186 (11.5%)	138,154 (11.5%)	32 (5.3%)	
>21 days	54,973 (4.6%)	54,435 (4.5%)	538 (89.5%)	
Mortality				
In-hospital mortality	14,797 (1.2%)	14,772 (1.2%)	25 (4.2%)	<0.001

**Table 4:** Univariate and multivariate analysis of risk factors for deep surgical site infection.

Variable	N	Univariate			Multivariate		
		OR	95% CI	*p*-Value	OR	95% CI	*p*-Value
Age (years)	123,833	0.98	0.97–0.98	<0.001	0.99	0.98–0.99	<0.001
Male sex	123,833	1.37	1.06–1.78	0.016	1.03	0.79–1.35	0.832
Injury severity score	123,833	1.08	1.07–1.08	<0.001	1.05	1.04–1.06	<0.001
Penetrating trauma	123,833	1.62	1.18–2.18	0.014	0.72	0.26–2.05	0.536
Mechanism of injury							
Motorcycle crash	123,833	1.03	0.63–1.59		1.32	0.80–2.04	0.249
Fall	123,833	0.13	0.09–0.20	<0.001	0.28	0.18–0.44	<0.001
Firearm	123,833	1.38	0.97–1.93		1.43	0.50–4.07	0.500
Other	123,833	0.43	0.26–0.67		0.58	0.26–1.13	0.141
Assault/Violence	123,833	1.39	0.99–1.92	0.150	0.89	0.41–1.96	0.761
Major liver injury	123,833	1.95	1.23–2.94	0.006	0.72	0.45–1.12	0.168
Major small bowel injury	123,833	3.07	1.57–5.34	0.002	0.83	0.41–1.52	0.567
Major kidney injury	123,833	2.09	0.99–3.82	0.052	1.06	0.50–1.97	0.868
Major liver resection	123,833	53.1	2.86–290	0.015	5.58	0.26–45.0	0.155
Major abdominal surgery	123,833	5.58	4.22–7.29	<0.001	3.08	2.21–4.23	<0.001
ICU length of stay (days)	123,833	1.05	1.05–1.06	<0.001	1.04	1.03–1.05	<0.001

## Data Availability

The data that support the findings of this study are available from the National Trauma Data Bank (NTDB), but restrictions apply to the availability of these data, which were used under license for the current study, and thus are not publicly available.
